# Systemic Inflammation Induces Acute Behavioral and Cognitive Changes and Accelerates Neurodegenerative Disease

**DOI:** 10.1016/j.biopsych.2008.07.024

**Published:** 2009-02-15

**Authors:** Colm Cunningham, Suzanne Campion, Katie Lunnon, Carol L. Murray, Jack F.C. Woods, Robert M.J. Deacon, J. Nicholas P. Rawlins, V. Hugh Perry

**Affiliations:** a Department of Biochemistry, Trinity College, Institute of Neuroscience, Trinity College Dublin, Republic of Ireland; b CNS Inflammation Group, Southampton Neurosciences Group, School of Biological Sciences, University of Southampton, Bassett Crescent East; c Department of Experimental Psychology, University of Oxford, South Parks Road, Oxford, United Kingdom

**Keywords:** Alzheimer's disease, cytokines, delirium, inflammation, microglial priming, neurodegeneration, systemic

## Abstract

**Background:**

Chronic neurodegeneration results in microglial activation, but the contribution of inflammation to the progress of neurodegeneration remains unclear. We have shown that microglia express low levels of proinflammatory cytokines during chronic neurodegeneration but are “primed” to produce a more proinflammatory profile after systemic challenge with bacterial endotoxin (lipopolysaccharide [LPS]).

**Methods:**

Here, we investigated whether intraperitoneal (IP) challenge with LPS, to mimic systemic infection, in the early stages of prion disease can 1) produce exaggerated acute behavioral (*n* = 9) and central nervous system (CNS) inflammatory (*n* = 4) responses in diseased animals compared with control animals, and 2) whether a single LPS challenge can accelerate disease progression (*n* = 34–35).

**Results:**

Injection of LPS (100 μg/kg), at 12 weeks postinoculation (PI), resulted in heightened CNS interleukin-1 beta (IL-1β), tumor necrosis factor-alpha (TNF-α), and interferon-beta (IFN-β) transcription and microglial IL-1β translation in prion-diseased animals relative to control animals. This inflammation caused exaggerated impairments in burrowing and locomotor activity, and induced hypothermia and cognitive changes in prion-diseased animals that were absent in LPS-treated control animals. At 15 weeks PI, LPS (500 μg/kg) acutely impaired motor coordination and muscle strength in prion-diseased but not in control animals. After recovery, these animals also showed earlier onset of disease-associated impairments on these parameters.

**Conclusions:**

These data demonstrate that transient systemic inflammation superimposed on neurodegenerative disease acutely exacerbates cognitive and motor symptoms of disease and accelerates disease progression. These deleterious effects of systemic inflammation have implications for the treatment of chronic neurodegeneration and associated delirium.

Chronic neurodegeneration is accompanied by an inflammatory response characterized by a selective activation of the microglial cells of the central nervous system (CNS) (1). This inflammation is assumed to contribute to disease progression through the production of inflammatory mediators. Although long-term nonsteroidal anti-inflammatory drug (NSAID) use modestly protects against the development of Alzheimer's disease (AD) (2), prospective studies with these drugs in AD patients have been disappointing (3–5). This emphasizes the considerable gaps in our knowledge about how microglial activation influences chronic neurodegeneration.

Systemic infections have more severe consequences in the elderly and demented population. Systemic infection is a common trigger for episodes of delirium (6–8), and episodes of delirium are predictive of increased morbidity and mortality (9–11). Surprisingly, direct investigations as to whether systemic inflammation accelerates the progression of dementia and neurodegenerative disease are lacking. Thus, whether and how systemic inflammation and CNS disease interact to accelerate neurodegeneration and to induce delirium is poorly understood.

It is well known that systemic inflammation, induced by endotoxin or proinflammatory cytokines, can induce CNS-mediated behavioral responses known collectively as sickness behavior (12). These include the fever response and decreased locomotor and reward-seeking activities and are driven by the CNS synthesis of inflammatory mediators. We have previously shown that sickness behavior responses to peripheral challenge with bacterial endotoxin (lipopolysaccharide [LPS]) are exaggerated in late-stage prion-diseased mice with chronic neurodegeneration and associated microglial “priming” (13). This priming consists of morphological and cell surface marker evidence of activation (14) but minimal proinflammatory cytokine synthesis (15–17). However, these primed hippocampal microglia undergo a phenotypic switch and produce a pronounced proinflammatory profile, characterized by microglial interleukin-1 beta (IL-1β), upon systemic challenge with LPS (13,18).

In the current study, we hypothesized that a systemic inflammatory episode induced by bacterial endotoxin (100 μg/kg) in animals with prion disease would induce exaggerated inflammatory and sickness behavioral responses and acute hippocampal-dependent cognitive impairment, in a novel shallow water Y-maze, that is absent upon similar challenge in normal animals or in saline-treated prion animals. We also hypothesized that a single systemic LPS challenge at 14 to 15 weeks postinoculation would induce acute impairments in motor coordination and muscle strength only in prion-diseased animals and would accelerate or exacerbate progression of the disease.

## Methods and Materials

### Animals and Stereotaxic Surgery

Female C57BL/6 mice, *n* = 233 (Harlan Olac, United Kingdom), were housed in cages of five at 21°C with a 12:12 hour light-dark cycle (lights on 0700 to 1900) with free access to food and water. They were anesthetized intraperitoneally (IP) with Avertin (Sigma, Poole, United Kingdom) and positioned in a stereotaxic frame. One microliter of a 10% wt/vol scrapie-infected (ME7 strain) C57BL/6 brain homogenate (or 10% wt/vol normal brain homogenate [NBH]) was injected into both dorsal hippocampi (from bregma: anterior-posterior −2.5 mm, lateral −1.7 mm, depth −1.6 mm) using a microsyringe (Hamilton, Reno, Nevada). Further animals (*n* = 9) were injected intracerebrally (IC) with *N*-methyl-D-aspartate (NMDA) (10 mg/mL) to ablate the hippocampus. This surgery was performed under isoflurane anesthesia (approximately 2%) with perisurgical analgesia (carprophen, 5 mg/kg). Chlordiazepoxide (CDZP; 10 mg/kg) and atropine (.075 mg/kg) were given to minimize seizure activity and bronchial secretions, respectively. In all other respects, the hippocampal lesions were performed as previously described (19). Sham-operated animals (*n* = 12) had eight holes drilled in the skull but no IC injections made. Female animals were used for all studies to minimize the occurrence of fighting, since this has significant effects on behavior.

### Intraperitoneal Challenges

Experimental groups at 12 weeks postinoculation with ME7 or NBH were injected with 100 μg/kg of LPS IP (Equine abortus [L5886], Sigma) in a volume of 200 μL saline. Prior studies demonstrated exacerbated neuronal death and floor effects on sickness behavior with LPS at 500 μg/kg (13). We thus injected 500 μg/kg to examine the longitudinal effect of LPS on disease progression at 14 to 15 weeks (*n* = 34–35) but injected 100 μg/kg to elucidate exaggerated sickness effects at 12 weeks (*n* = 9). ME7 animals were given lower doses of LPS than NBH animals in Y-maze experiments (100 μg/kg vs. 500 μg/kg, *n* = 23–24) as a conservative measure, such that ME7 + LPS animals were not more sick than the NBH + LPS animals (to verify that any cognitive change observed was not attributable to some nonspecific aspect of exaggerated sickness). This was verified by open field activity assessment (data not shown). Control animals were administered 200 μL nonpyrogenic saline.

### Burrowing

Mice were placed in individual cages with a food pellet-filled, opaque plastic burrow tube as previously described (20). The weight of pellets displaced in a 2-hour period was measured for several baseline sessions prior to LPS administration. On the day of LPS challenge, mice were placed in the cages to measure burrowing 2 hours after LPS and also at 24 and 48 hours later.

### Open Field Activity

Open field activity was assessed using activity monitor software (Med Associates Inc., Georgia, Vermont) as previously described (21). The open field consisted of an aluminium base (27 × 27cm) enclosed on four sides with a .7-cm-thick clear acrylic sheet, surrounded by an opaque screen. Animals were tested at weekly intervals from 6 weeks postinoculation, thus ensuring baseline stability. Locomotor activity was measured immediately before LPS challenge and then 5 hours and 24 hours following IP challenges.

### Body Temperature

A thermocouple rectal probe (Thermalert TH5, Physitemp, Clifton, New Jersey) was used to measure core body temperature. The mice were preadapted to measurements of rectal temperature for 2 days prior to the IP challenges to minimize stress effects. Temperatures were taken at baseline and then at 5 hours and 24 hours postchallenge (approximately 5:00 pm and 11:00 am).

### Spatial Learning

We have developed a novel “paddling” Y-maze task to investigate cognitive status in sick animals since reduced appetitive, motivational, and locomotor drives confound data from many common tests of hippocampal function. This Y-maze task has recently been published (22) and combines elements of two hippocampal-dependent tasks: the paddling pool spatial cognition test and the appetitive Y-maze (23,24). Briefly, a clear perspex Y-maze mounted on a white plastic base (constructed in the School of Biomedical Sciences, University of Southampton) was filled with 2 cm of water at 20°C to 22°C, sufficient to motivate mice to leave the maze by paddling to an exit tube at the distal end of one arm, 2 cm above the floor. The mouse exits to a burrowing tube in which it is returned to its home cage. Burrowing tubes were also placed over the false exit tubes so that, from the center, all arms looked identical. The maze was placed in the middle of a small room surrounded by prominent visual cues. The location of the exit was fixed for each animal. Mice were placed in one of the two possible start arms in a pseudorandomized sequence for 10 trials and the groups were counterbalanced with respect to the location of the exit. ME7 and NBH mice were treated with saline or LPS and assessed, starting 3 hours postchallenge, on incorrect trials (failure to choose the correct arm first) and total number of arms entered. An arm entry was defined as entry of the whole body, excluding the tail. Mice with NMDA lesions of the hippocampus and sham-operated animals were also tested to verify hippocampal dependence of the task.

### Motor Tests: Horizontal Bar and Inverted Screen

The horizontal bar assessed forelimb muscular strength and coordination. It consisted of a 26 cm long metal bar, .2 cm diameter, supported by a 19.5 cm high column at each end. Each mouse was held by the tail, placed with its front paws at the central point of the bar, and rapidly released. A time was assigned depending on whether and when the mouse fell, held on for 60 seconds, or reached a supporting column (the latter two results scoring the maximum of 60 seconds). The inverted screen (25) assessed muscular strength for all four limbs. It consisted of a wooden frame, 43 cm square, covered with wire mesh (12 mm squares of 1 mm diameter wire). The mouse was placed on the screen, which was then slowly (2 seconds) inverted. The time it took for the mouse to fall was measured, up to a criterion time of 60 seconds. Padding was provided to cushion mice falling off either apparatus.

### Quantitative PCR

Animals were terminally anesthetized and transcardially perfused with heparinized saline 2 hours postadministration of LPS or saline. Brains were rapidly removed and the hippocampi were dissected out, snap frozen in liquid nitrogen, and stored at −80°C. Total RNA was extracted using Qiagen RNeasy mini columns (Qiagen, Crawley, United Kingdom) according to the manufacturer's instructions. Contaminating genomic DNA was degraded during extraction with Qiagen DNase1 (Qiagen). Two hundred nanograms of total RNA were reverse-transcribed in a 10 μL reaction volume and 1 μL of the reverse transcription (RT) reaction was used for polymerase chain reaction (PCR). Equipment and reagents for quantitative PCR were supplied by Applied Biosystems (Warrington, United Kingdom). Assays for IL-1β, tumor necrosis factor-alpha (TNF-α), and interferon-beta (IFN-β) were designed using the published sequences for these genes and Primer Express software (Applied Biosystems, Warrington, United Kingdom) and quantified using a relative standard curve. These methods and primer sequences have been described elsewhere (26) and are included in full in Supplement 1. All PCR data were normalized to the expression of the housekeeping gene glyceraldehyde-3-phosphate dehydrogenase (GAPDH).

### Immunohistochemistry

Immunohistochemistry for cyclooxygenase-2 (COX-2) and IL-1β was carried out on formalin-fixed, paraffin-embedded sections. Sections were dewaxed, rehydrated, quenched with 1% hydrogen peroxide (H_2_O_2_) in absolute methanol, and washed briefly in phosphate buffered saline (PBS) before antigen retrieval by microwaving in citrate buffer (pH 6) for 2 × 5 minutes. Sections were blocked with 10% horse serum (for COX-2) or 20% normal goat serum (for IL-1β) before overnight incubation with the primary antibody at 1/1000 (goat polyclonal anti-COX-2, Santa Cruz Biotechnology Inc., Santa Cruz, California) or 1/50 (rabbit polyclonal anti-IL-1β, Peprotech, London, United Kingdom) prepared in 20% serum to block nonspecific interactions. The sections were washed and then incubated with the appropriate biotinylated secondary antibody. Labeling was visualized using the avidin-biotin-peroxidase complex (ABC method, using .015% vol/vol hydrogen peroxide as substrate and diaminobenzidine as chromagen (Sigma) as previously described (26).

### Statistical Analyses

Acute biochemical experiments were analyzed by one-way analysis of variance (ANOVA) with Bonferroni post hoc comparisons. Interaction between disease and acute treatment was analyzed using two-way ANOVA. All acute behavioral experiments were analyzed using a planned comparison by one-way ANOVA, with Bonferroni post hoc tests, at the time predicted to produce clearest differences in LPS-induced effects. Parametricity was tested by Kolmogorov-Smirnov test. Horizontal bar and inverted screen data were nonparametric and were analyzed by Kruskal-Wallis at 6 hours post-LPS challenge and across the post-LPS time course by repeated measures two-way ANOVA. The numbers reaching a criterion of 60 seconds were also compared by one-tailed Fisher exact test, with Tocher's modification. Median numbers of errors made on Y-maze acquisition were compared by the Kruskal-Wallis one-way ANOVA with Dunn post hoc tests and also analyzed by repeated measures ANOVA to assess acquisition across two blocks of trials. Longitudinal effects of LPS on disease progression were assessed by repeated measures ANOVA.

## Results

### Acute LPS-Induced Changes at 12 Weeks

At 12 weeks postinoculation, ME7 and NBH mice showed equivalent burrowing and open field performance prior to LPS challenge. All treatment groups burrowed, on average, between 150 g and 170 g of pellets in baseline 2-hour sessions. Average baseline distance travelled was between 240 cm and 330 cm for all groups.

### Burrowing

**Figure 1:**
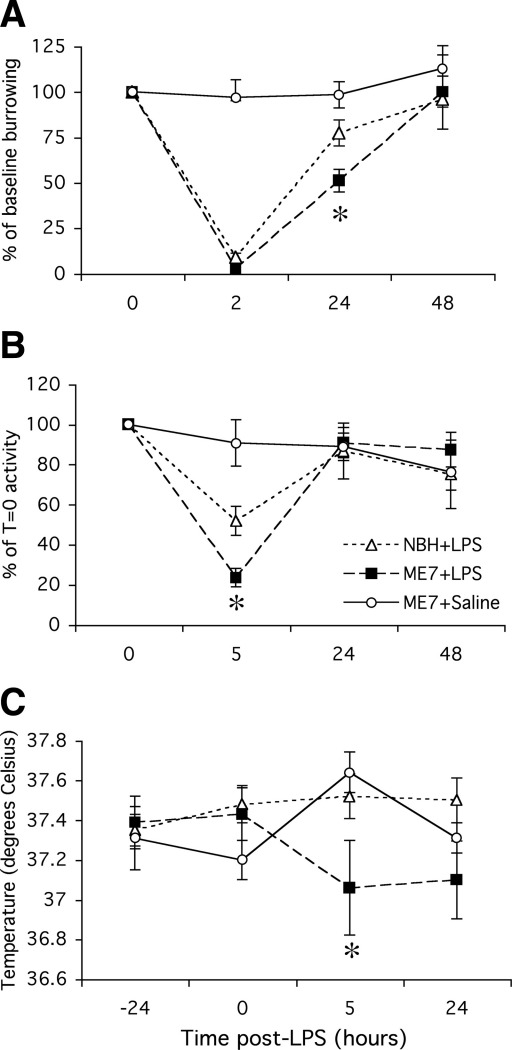
Acute sickness behavioral responses to intraperitoneal LPS. **(A)** Weight burrowed over 2 hours by C57BL/6 mice at baseline, 2 hours, 24 hours, and 48 hours post-IP challenge with 100 μg/kg LPS or sterile saline. ME7 and NBH animals were 12 weeks postinoculation with the ME7 murine scrapie strain or normal brain homogenate, respectively. Statistical significance was determined by planned comparisons by one-way ANOVA with Bonferroni post hoc test at 24 hours. **(B)** Distance traveled in an open field by the same animals at baseline, 5 hours, 24 hours, and 48 hours postchallenge with LPS. Statistical significance was determined by planned comparisons by one-way ANOVA with Bonferroni post hoc test at 5 hours. **(C)** Core body temperature of the same animals at −24, 0, 5, and 24 hours postchallenge with LPS or saline. Planned comparison by one-way ANOVA at 5 hours was performed. All data are plotted as mean ± SEM and *n* = 9 in all groups. *p < .05. ANOVA, analysis of variance; IP, intraperitoneal; LPS, lipopolysaccharide; NBH, normal brain homogenate.

Between 2 and 4 hours postsystemic challenge with LPS, all LPS-treated animals showed complete inhibition of burrowing (Figure 1A). At 24 hours, there was good recovery of burrowing in NBH + LPS animals (not significantly different from that at 0 time [baseline burrowing taken 24 hours before LPS challenge, *p* > .05) but ME7 + LPS animals were still impaired to approximately 50% (one-way ANOVA, *p* < .05). All animals returned to normal by 48 hours. Thus, systemic inflammatory challenge induced a greater inhibition of burrowing in prion-diseased than in control animals.

### Open Field Activity

Lipopolysaccharide (100 μg/kg) reduced locomotor activity in both normal NBH and prion-diseased mice (Figure 1B) but this inhibition was greater (approximately 80% vs. 50%) in ME7 animals than NBH control animals (*p* < .05, one-way ANOVA with Bonferroni post hoc test at 5 hours post-LPS). Normal open field activity had recovered in all groups by 24 hours.

### Body Temperature

It is known that LPS causes hypothermia rather than hyperthermia in the C57BL/6 mouse at ambient temperatures (27). However, small changes in body temperature are difficult to detect using rectal probe measurements. Accordingly, LPS did not induce a measurable hypothermia at 5 hours in NBH control animals (Figure 1C). In contrast, a clear and statistically significant hypothermic response was observed in ME7 animals given the same dose of LPS (*p* < .05, one-way ANOVA with Bonferroni post hoc test at 5 hours post-LPS). Thus, LPS at 100 μg/kg induces a measurable hypothermia in ME7 but not in NBH animals.

### Inflammatory Changes at 12 Weeks

**Figure 2:**
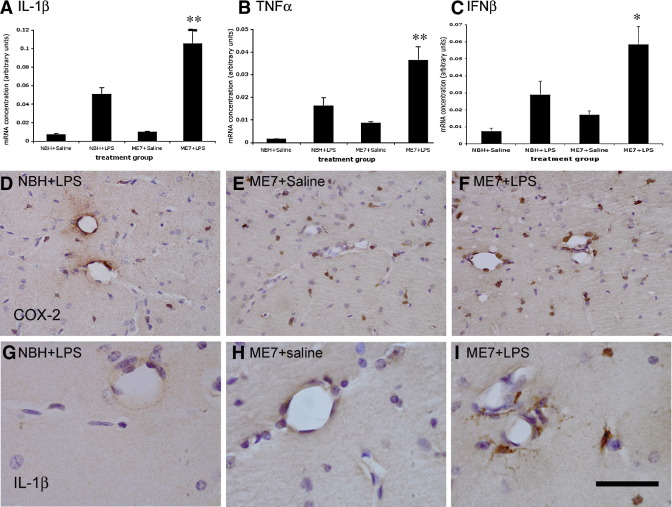
Inflammatory changes post-LPS at 12 weeks. Relative mRNA expression for treatment groups NBH + saline, NBH + LPS 100 μg/kg, ME7 + saline, and ME7 + LPS 100 μg/kg for **(A)** IL-1β, **(B)** TNF-α, and **(C)** IFN-β. In all cases, *n* = 4 for all groups except ME7 + saline (*n* = 5). Significant interactions of disease and acute treatment by two-way ANOVA are denoted by two asterisks (**) (IL-1β *p* < .0001; TNF-α *p* = .0013) and a significant difference between ME7 + LPS and all other groups by one-way ANOVA is denoted by one asterisk (*). **(D)** COX-2 staining (brown) both around the vasculature and in the parenchyma after IP challenge of NBH animals with LPS. **(E)** Increased COX-2 positive cells, with respect to NBH, in the parenchyma of ME7 animals treated with saline. **(F)** COX-2 positive stained cells in both the parenchyma and around the vasculature of ME7 animals challenged with LPS. **(G-I)** IL-1β positive staining in microglial cells in the hippocampus of ME7 animals challenged IP with LPS **(I)** compared with an absence of this staining in ME7 per se **(H)** and in NBH animals treated with LPS **(G)**. Scale bar represents 100 μm **(D, F)** and 40 μm **(G-I)**. ANOVA, analysis of variance; COX-2, cyclooxygenase-2; IFN-β, interferon-beta; IL-1β, interleukin-1 beta; IP, intraperitoneal; LPS, lipopolysaccharide; mRNA, messenger RNA; NBH, normal brain homogenate; TNF-α, tumor necrosis factor-alpha.

Expression of messenger RNA (mRNA) for the genes IL-1β, TNFα, and IFN-β was observed 2 hours after challenge of NBH animals with LPS (100 μg/kg). Bonferroni post hoc tests after a significant one-way ANOVA showed that the magnitude of this increase, for all three genes, was significantly higher in ME7 animals given the same LPS challenge (Figure 2A–C): NBH + LPS versus ME7 + LPS, *p* < .001 (IL-1β), *p* < .01 (TNFα), and *p* < .05 (IFN-β). A two-way ANOVA analysis revealed that there were, as expected, clear main effects of LPS on all three genes. However, there was also a significant interaction of disease and acute treatment on expression of IL-1β [*F*(3,13) = 70.49, *p* < .0001] and TNFα [*F*(3,13) = 16.76, *p* = .0013], whereas that for IFN-β was not quite significant [*F*(3,13) = 3.54, *p* = .0825]. Microglial activation was apparent in all ME7 animals as evidenced by increased numbers of microglial cells positively stained for COX-2 in the hippocampus compared with that in NBH animals (Figure 2D–F). As previously observed (18), COX-2 staining was also observed constitutively in perivascular macrophages and inducibly in endothelial cells after LPS challenge. Phenotypic switching of microglia to the more proinflammatory state is manifest as increased IL-1β staining in ME7 + LPS animals compared with NBH + LPS animals, which do not show detectable microglial IL-1β staining (Figure 2G-I). Interleukin-1 beta positive microglia were found proximal to hippocampal blood vessels, but we cannot rule out the possibility of IL-1β that is below the limit of detection in NBH + LPS animals or deeper into the parenchyma of ME7 + LPS animals.

### Spatial Reference Memory in the Y-Maze

**Figure 3:**
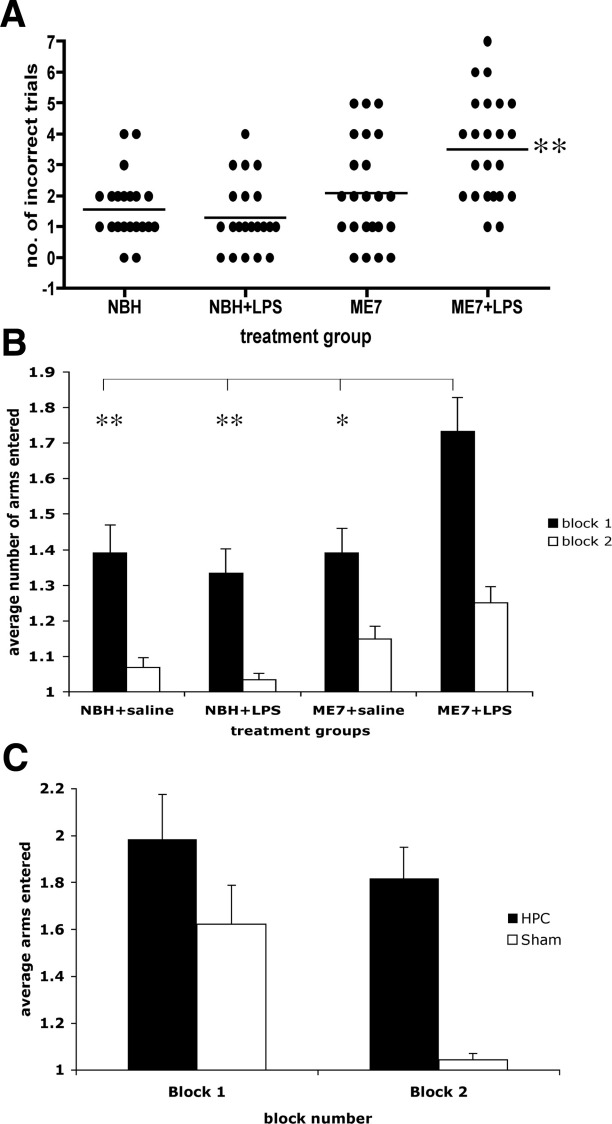
Spatial learning in the paddling Y-maze: normal and prion-diseased animals and hippocampal-lesioned and sham-operated control animals. **(A)** Cognitive performance of animals in the paddling Y-maze at 12 weeks postinoculation with ME7 (100 μg/kg LPS or saline) and NBH (500 μg/kg LPS or saline) 3 to 7 hours after IP challenge. This plot represents the total number of incorrect trials (out of 10) by each treatment group. Each animal is shown as an individual point and the median is illustrated by the bar. Kruskal-Wallis ANOVA revealed a difference in the medians (*p* < .0001) and pairwise comparisons showed ME7 + LPS to be significantly different to all other groups (**), *n* = 23 in the case of both NBH groups and *n* = 24 for both ME7 groups. **(B)** The same data are plotted as two successive blocks of five trials, showing the average number of arms entered including the final correct one. Block 2 is compared with block 1 by Student *t* tests in all cases (*p < .05). The average number of arms entered in block 1 by each group was also compared by ANOVA with Bonferroni post hoc test (*p < .05, **p < .01). **(C)** Hippocampal-lesioned animals compared with sham-operated control animals tested across two blocks of five trials on the same paddling Y-maze. A repeated measures ANOVA analysis shows a significant effect of treatment (*F* = 10.78, *p* = .0039, *df* = 1,19) and of trial (*F* = 3.54, *p* = .0005, *df* = 9,171). ANOVA, analysis of variance; IP, intraperitoneal; LPS, lipopolysaccharide; NBH, normal brain homogenate.

Normal brain homogenate and ME7 animals were treated with saline or LPS (*n* = 23 for NBH, *n* = 24 for ME7) and tested in the visuospatial reference memory paddling Y-maze, between 3 and 7 hours, to test for LPS-induced learning impairments. The ME7 animals challenged with LPS made more incorrect arm entries across 10 trials than any of the other groups (Figure 3A; *p* < .0001 Kruskal-Wallis test). Dunn's multiple comparison post hoc tests revealed ME7 + LPS animals to be significantly different from all other groups (*p* < .05). Plotted as the average number of arms entered, in two blocks of five trials (Figure 3B), ME7 + LPS animals made considerably more errors in block 1 and this persisted to a lesser degree in block 2. These animals were hypoactive at this time, so increased arm entries cannot be explained by increased activity. Despite the sickness of the NBH + LPS animals, their learning was not significantly impaired compared with NBH + saline animals (Figure 3B). This simple Y-maze has been designed to allow learning to be achieved in a single session, while LPS effects persist, to eliminate state-dependent effects. Thus, comparison of block 2 with block 1 (paired *t* tests *p* < .0013) is suggestive of some learning in all groups. Repeated measures ANOVA comparison of arm entries by ME7 + LPS and ME7 animals shows main effects of treatment (*F* = 9.66, *p* = .0032, *df* = 1,46) and of trial (*F* = 9.9, *p* < .0001, *df* = 9,414) but that for NBH + saline and NBH + LPS shows an effect of trial (*F* = 24.88, *p* < .0001, *df* = 9,396) but no effect of treatment (*F* = 1.62, *p* = .2091, *df* = 1,44). Thus, LPS has no impact on the accuracy of NBH animals in this maze task but does impair that of ME7 animals.

Acquisition of correct responding in this maze was hippocampal-dependent (Figure 3C). Sham-operated animals quickly learned the location of the exit while hippocampal-lesioned animals continued responding at chance levels (50%). A repeated measures ANOVA analysis shows a significant effect of treatment (*F* = 10.78, *p* = .0039, *df* = 1,19).

### Acute LPS-Induced Changes at 15 Weeks

#### Horizontal Bar and Inverted Screen

**Figure 4:**
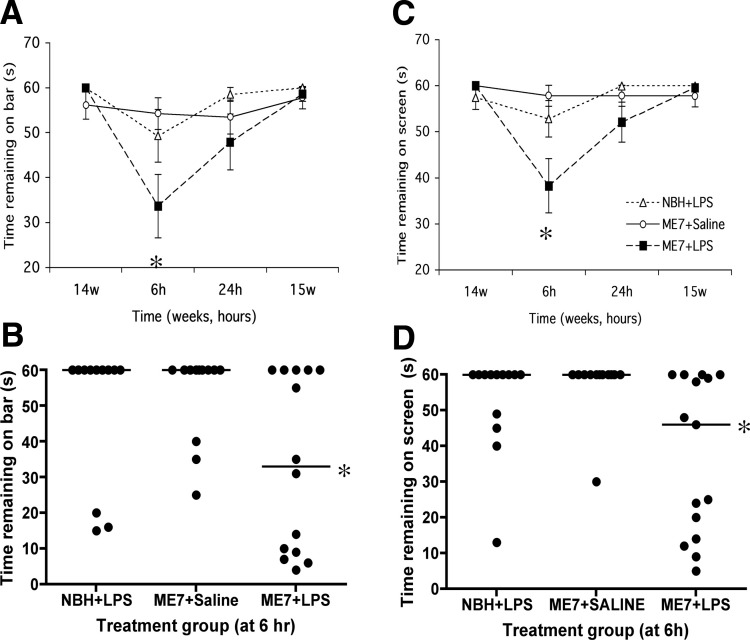
Acute effects on motor tasks induced by LPS in normal and prion-diseased animals. **(A)** Performance on a horizontal bar at 14 weeks postinoculation with ME7 or NBH, followed by performance at 6 hours, 24 hours, and 1 week after IP LPS (500 μg/kg) or saline challenges in these animals. Data were plotted as mean ± SEM and analyzed by repeated measures ANOVA Bonferroni post hoc tests (*p* < .05 between ME7 + LPS and NBH + LPS, *n* = 13–15 for all groups). **(B)** Replot of the 6-hour post-LPS time point showing the median and full range of values. These data were compared by Kruskal-Wallis test with Dunn's post hoc test (*p < .05). **(C)** Performance on an inverted screen at the same time points as described above. Data are shown as mean ± SEM and were analyzed by repeated measures ANOVA with Bonferroni post hoc test (*p* < .001 between ME7 + LPS and NBH + LPS, *n* = 13–15 for all groups). **(D)** Replot of the inverted screen 6-hour post-LPS time point showing the median and full range of values. These data were compared by Kruskal-Wallis test with Dunn's post hoc test (*p < .05). ANOVA, analysis of variance; IP, intraperitoneal; LPS, lipopolysaccharide; NBH, normal brain homogenate.

All animal groups performed the horizontal bar task well at 14 weeks (Figure 4A). Upon IP challenge with LPS, ME7 animals showed a decrease in performance relative to the NBH + LPS group. Repeated measures ANOVA reveals an interaction of time and treatment (*F* = 3.05, *p* = .0085, *df* = 6,114) and Bonferroni post hoc test at 6 hours reveals significant impairment of ME7 + LPS (*p* < .05). ME7 + saline animals performed well throughout the experiment. Since the data were nonparametric, a scatter dot plot is also shown (Figure 4B). Nine out of 12 NBH + LPS animals reached a criterion of 60 (median = 60), while 9 out of 14 ME7 + LPS animals failed to reach this criterion (median = 38). The proportions of each group that reached criterion are significantly different by one-tailed Fisher exact test with Tocher's modification. Furthermore, when the times are compared by Kruskal-Wallis, with a Dunn post hoc test, ME7 + LPS and NBH + LPS are significantly different (*p* < .05).

All animal groups showed good performance on the inverted screen at 14 weeks (Figure 4C). Lipopolysaccharide induced a clear decrease in times in ME7 animals compared with the same challenge in NBH animals and to saline challenges in ME7 animals (Bonferroni post hoc test at 6 hours, *p* < .001). This decrease is most obvious at 6 hours but is still apparent at 24 hours and repeated measures ANOVA reveals a main effect of treatment (*F* = 5.35, *p* = .0088, *df* = 2,39) and an interaction of time and treatment (*F* = 4.49, *p* = .0004, *df* = 6,117). We also provide scatter dot plots of the 6-hour data (Figure 4D). When these data are compared by Kruskal-Wallis, with Dunn post hoc tests, there is a statistically significant difference between ME7 + LPS and NBH + LPS (*p* < .05).

All groups showed good recovery on both tasks by 15 weeks. These data demonstrate a greater acute effect of LPS on motor coordination and muscle strength in ME7 animals than in NBH control animals. When administered at 12 weeks postinoculation with prion disease, LPS did not have any acute effects on motor coordination or muscle strength (data not shown).

### Longitudinal Effects of Systemic Infection on Disease Progression

**Figure 5:**
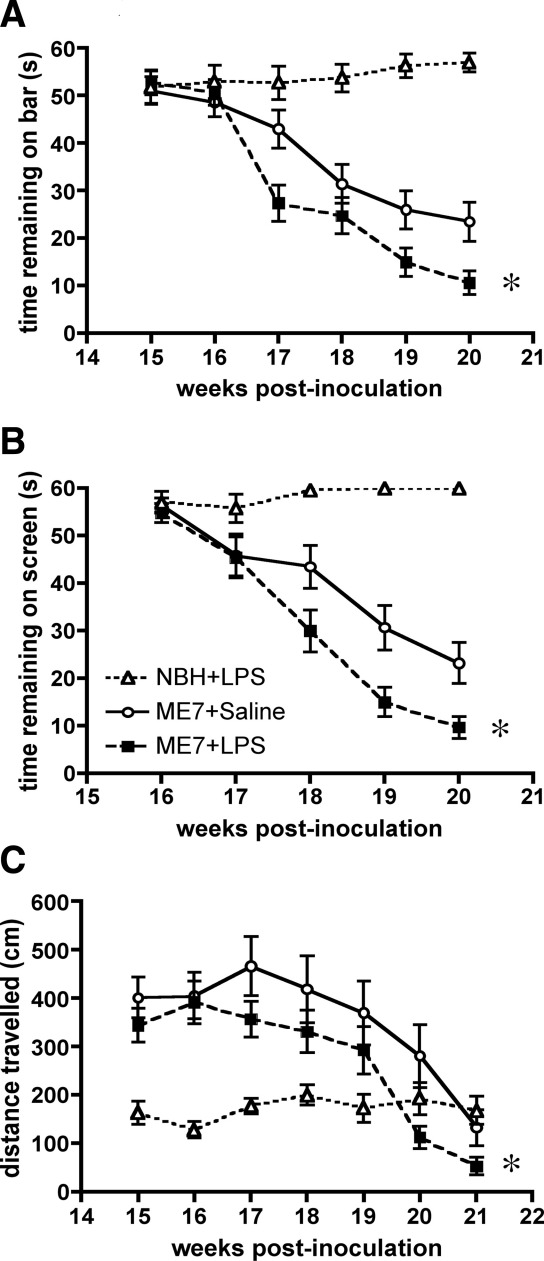
Longitudinal effects of LPS at 14 to 15 weeks on the progression of prion disease. **(A)** Performance on the horizontal bar for 6 successive weeks post-IP challenge with LPS (500 μg/kg) or saline at 15 weeks postinoculation with ME7 or NBH. Data are shown as mean ± SEM (*n* = 25 for NBH + LPS, *n* = 34 for ME7 + Saline, and *n* = 35 for ME7 + LPS). ME7 + LPS and ME7 + saline data were compared by repeated measures ANOVA across the 6 weeks shown and showed both main effects of treatment and time and an interaction of treatment and time (* denotes *p* = .0166 for the main effect of treatment). **(B)** Performance of the same animals on the inverted screen for 5 successive weeks post-IP challenge with LPS (500 μg/kg) or saline. Data are shown as mean ± SEM (*n* = 25 for NBH + LPS, *n* = 34 for ME7 + Saline, and *n* = 35 for ME7 + LPS). ME7 + Saline and ME7 + LPS data were compared by repeated measures ANOVA across the 5 weeks shown and showed both main effects of treatment and time and an interaction of treatment and time (** denotes *p* = .0147 for the main effect of treatment). **(C)** Open field activity for 7 successive weeks post-IP challenge with LPS 500 μg/kg. Data are shown as mean ± SEM (*n* = 19, *n* = 20). Repeated measures ANOVA of ME7 groups showed main effects of time and treatment (* denotes *p* = .0044 for the main effect of treatment). ANOVA, analysis of variance; IP, intraperitoneal; LPS, lipopolysaccharide; NBH, normal brain homogenate.

As previously shown, there is a clear impairment in performance on the horizontal bar and the inverted screen in the ME7 groups between 16 and 20 weeks (Figure 5). ME7 animals challenged with 500 μg/kg LPS between 14 and 15 weeks subsequently showed more impairment on the horizontal bar than ME7 + saline animals, and their performance on this task remained worse throughout the experiment (Figure 5A). A repeated measures ANOVA analysis of the two ME7 groups revealed main effects of treatment (*F* = 6.04, *p* < .05, *df* = 1,65) and of time (*F* = 43.77, *p* < .0001, *df* = 4,260) and an interaction of treatment and time (*F* = 3.06, *p* < .05, *df* = 4,260).

ME7 animals challenged with 500 μg/kg LPS between 14 and 15 weeks also subsequently showed an earlier onset of impairment on the inverted screen than ME7 + saline animals, and this performance remained worse than that for ME7 + saline animals throughout the experiment (Figure 5B). A repeated measures ANOVA analysis of the two ME7 groups revealed main effects of treatment (*F* = 6.41, *p* < .05, *df* = 1,47) and of time (*F* = 53.86, *p* < .0001, *df* = 4,188) and an interaction of treatment and time (*F* = 2.76, *p* < .05, *df* = 4,188).

ME7 animals challenged with LPS 500 μg/kg between 14 and 15 weeks also subsequently showed an earlier decrease in open field locomotor hyperactivity. Hyperactivity of both ME7 groups relative to NBH control animals is clear in Figure 5C, but this is less marked in the ME7 + LPS group, suggesting the beginning of the late-stage decline. Repeated measures ANOVA analysis of the two ME7 groups shows a main effect of time (*F* = 12.86, *p* < .0001, *df* = 6,228) and a main effect of treatment (*F* = 9.15, *p* = .0044, *df* = 1,38).

All of these LPS-induced decrements are temporally separated from the acute LPS effects on these tasks shown in Figure 4 (A,B) and together these data demonstrate that a single, transient, peripheral inflammatory event can hasten the disease process, accelerating the appearance of three different neurological signs of disease.

## Discussion

In the current study, we have shown that systemic LPS during presymptomatic chronic neurodegenerative disease can induce exaggerated CNS inflammation and sickness behavior responses compared with those in normal animals. Acute symptoms also now extend beyond the typical spectrum of sickness behavior responses to include exacerbation of symptoms more commonly associated with the underlying disease: acute cognitive impairments (LPS at 12 weeks) and acute impairments of motor coordination and muscle strength (LPS at 15 weeks). In addition, a single LPS challenge accelerates and exacerbates the progression of the underlying disease. Thus, on multiple levels, systemic inflammation impacts negatively on chronic neurodegeneration.

### Exaggerated Sickness Behavior and Microglial Priming

We had previously shown that at 18 weeks postinoculation with the ME7 strain of prion disease, these animals were susceptible to exaggerated sickness behavior responses to LPS (13). Such responses were absent at 8 weeks postinoculation, before large numbers of microglia become activated. Thus, microglia that are activated by the process of neurodegeneration (18,28) or aging (29,30) but which do not synthesize robust levels of proinflammatory cytokines are susceptible to phenotypic switching by systemic inflammatory insults. We demonstrated IL-1β synthesis specifically in ME7 hippocampal microglial cells after systemic LPS challenge but not in NBH + LPS animals or in ME7 animals injected with saline, despite marked neurodegeneration and extracellular prion protein (PrP^Sc^) deposits in the latter (18). In the current study, we have shown that this phenotypic switching, heightened cytokine production, and exaggerated sickness behavior are also induced at 12 weeks postinoculation with prion disease in the absence of neuronal loss but at the onset of hippocampal CA1 synaptic loss and hippocampal-dependent impairments (31,32). This, therefore, places subjects at risk of disease exacerbation from an early or even presymptomatic stage of disease. Whether the switch from the primed state to that expressing IL-1β is key to this disease exacerbation remains to be established, but given the high sensitivity of IL-1 type I receptors (33), neuroanatomically defined, microglial IL-1β protein expression constitutes a biologically significant event.

### Acute Cognitive Impairments

Lipopolysaccharide induced acute cognitive impairments in ME7 animals but not in NBH animals. Primed microglial cells are limited to the hippocampus and limbic system at this stage of disease (18,31), and therefore we hypothesized that hippocampal-dependent tasks would be sensitive to these insults. We have shown here that correct responding in the Y-maze spatial reference memory task is hippocampal-dependent. Thus, we have induced an acute memory deficit in LPS-treated ME7 animals that is hippocampal-dependent and is absent in LPS-treated NBH animals, despite obvious sickness behavior. Lipopolysaccharide is reported to affect cognitive function in young and aged mice (34–37), but there are potential confounding factors in these studies, directly referred to by the authors. The novel shallow water Y-maze task developed here assesses incorrect responses rather than time taken to solve the maze, thus minimizing such confounding factors. The cognitive impairment that we describe upon acute inflammatory insult during chronic neurodegeneration and similar impairments reported in aged animals challenged with LPS (38,39) have parallels with the acute cognitive impairments seen in episodes of delirium during dementia. Delirium is an acute and transient confusional state that commonly occurs after infection, surgery, or injury (40). Delirium is remarkably prevalent in the aged and demented population: 10% of over 65s admitted to the emergency room (41) and 22% to 89% in demented patients (42). The etiology of delirium is unknown and given its very high frequency in aged and demented populations, it is a major research question. The novel Y-maze easily distinguishes between sham and hippocampal-lesioned animals. Larger group sizes are required to distinguish between ME7 and ME7 + LPS animals, reflecting interanimal variability in the onset of cognitive changes in ME7 animals. This mirrors the difficulties in distinguishing chronic and acute impairments in dementia and delirium. We propose that further refinement of the model system used here will be useful in the study of how systemic inflammation and underlying dementia interact to produce these episodes.

### Acute Exacerbation Leads to Acceleration of Chronic Disease

Dementia is the single biggest risk factor for episodes of delirium and systemic inflammation is a major trigger in these patients. It is also now clear that a single episode of delirium is predictive of an accelerated rate of clinical decline in demented patients (9–11). This implies that the insults that induce episodes of delirium are also responsible for an acceleration of the disease process. We previously showed that a single challenge with LPS was sufficient to exacerbate neuronal death during prion disease (18). Repeated challenges with LPS have been shown to exacerbate axonal damage and disease progression in a transgenic model of amyotrophic lateral sclerosis (43). Similarly, there is evidence for exacerbation of features of pathology in a triple transgenic model of Alzheimer's disease (44) and for exacerbation of inflammation (45) and amyloid deposition (46) in AD models. In the current study, we have shown that a single challenge with LPS is sufficient to accelerate and exacerbate permanent loss of neurological function. We observed acute impairments of motor function during the systemic inflammatory episode, recovery of normal function postinflammation, and accelerated onset of progressive and permanent neurological changes. This mirrors the accelerated cognitive decline of demented patients after recovery from acute symptoms of delirium (47) and indicates that systemic inflammatory insults accelerate disease progression. It is of note that LPS administered at 12 weeks postinoculation with prion disease did not affect motor coordination, consistent with the idea that as pathology becomes more global, later in disease, the range of functions that may be affected by systemic inflammation becomes broader. It is of interest to examine whether pathology and behavioral symptoms in Tg2576 and CRND8 mice are similarly affected by systemic inflammation. However, that our hypothesis and the current model have validity in human neurodegenerative disease has been demonstrated in recent and ongoing clinical studies: carer-reported systemic infection and elevated concentrations of IL-1β in sera were independently associated with accelerated cognitive decline in Alzheimer's disease patients (48).

### Conclusion

We have shown a clear systemic inflammation-induced exacerbation of neurodegenerative disease. The mechanism of this exacerbation remains unclear. It is well known that peripheral inflammation can activate CNS centers by a number of routes, including the circumventricular organs, vagal afferents, and the brain endothelium (see 12 for review). Bacterial endotoxin can directly activate the brain endothelium (49,50), but it is well known that behavioral and CNS inflammatory changes can be induced by systemic administration of proinflammatory cytokines (12). Furthermore, it has recently been shown that systemic IL-1β exacerbates neurodegeneration and motor symptoms in a rat model of Parkinson's disease (51). Due to the large numbers of animals required to overcome interindividual variability in the current study, mechanistic analysis was impractical. However, preliminary investigations in normal animals show that indomethacin does not inhibit CNS proinflammatory cytokine transcription despite clear effects on LPS-induced behavioral changes and on prostaglandins (52). There remain many unanswered questions about the nature of the interactions between systemic and CNS inflammatory compartments in this model and in human disease. Studies addressing these interactions and the relative roles of endotoxin, cytokines, and prostaglandins will have implications for our understanding of the role of inflammation in neurodegenerative disease and in episodes of delirium.
