# Supplementary material for: Systemic Inflammation Induces Acute Behavioral and Cognitive Changes and Accelerates Neurodegenerative Disease
Source: Biol Psychiatry. 2009 Feb 15;65(4):304–12. doi: 10.1016/j.biopsych.2008.07.024 (PMC2633437; doi:10.1016/j.biopsych.2008.07.024)
Supplement: Supplement 1 [file mmc1.doc]

# Supplementary material

Standard curve construction for quantitative PCR

A standard curve was constructed from total RNA isolated from mouse brain tissue 24 hours after intra-cerebral challenge with 2.5 μg LPS, which is known to up-regulate all target transcripts of interest in this study. This standard curve was constructed using a higher concentration of RNA in the reverse transcriptase reaction than that in the samples for analysis to ensure that the cytokine transcription of all experimental animal groups will fall within the range of the standard curve constructed. Serial 1 in 5 dilutions of the cDNA synthesized from brains of LPS-injected mice were made and a curve plotted of the Ct value (the cycle number at which the fluorescence of the product of transcribed gene crosses the threshold of detection) versus the log of the concentration (assigned an arbitrary value since the absolute concentration of cytokine transcripts is not known). Verification that this curve is a straight line confirms the efficiency of the PCR reaction across the entire concentration range and the equation of this line can then be used to calculate the relative concentrations of the experimental samples. Since the top standard has been assigned an arbitrary value, it follows that the calculated concentrations of all experimental samples will also have arbitrary values, but these values will be directly related to the equation of the line.
